# Investigation of Preparation and Mechanisms of a Dispersed Particle Gel Formed from a Polymer Gel at Room Temperature

**DOI:** 10.1371/journal.pone.0082651

**Published:** 2013-12-06

**Authors:** Guang Zhao, Caili Dai, Mingwei Zhao, Qing You, Ang Chen

**Affiliations:** 1 State Key Laboratory of Heavy Oil Processing, China University of Petroleum, Qingdao, Shandong, P. R. China; 2 School of Energy Resources, China University of Geosciences, Beijing, P. R. China; University of Kansas, United States of America

## Abstract

A dispersed particle gel (DPG) was successfully prepared from a polymer gel at room temperature. The polymer gel system, morphology, viscosity changes, size distribution, and zeta potential of DPG particles were investigated. The results showed that zirconium gel systems with different strengths can be cross-linked within 2.5 h at low temperature. Scanning electron microscopy (SEM), transmission electron microscopy (TEM), and atomic force microscopy (AFM) results showed that the particles were polygonal particles with nano-size distribution. According to the viscosity changes, the whole preparation process can be divided into two major stages: the bulk gel cross-linking reaction period and the DPG particle preparation period. A polymer gel with a 3-dimensional network was formed in the bulk gel cross-linking reaction period whereas shearing force and frictional force were the main driving forces for the preparation of DPG particles, and thus affected the morphology of DPG particles. High shearing force and frictional force reduced the particle size distribution, and then decreased the zeta potential (absolute value). The whole preparation process could be completed within 3 h at room temperature. It could be an efficient and energy-saving technology for preparation of DPG particles.

## Introduction

Long-term water flooding during the development of oilfields has resulted in aggravated heterogeneity of reservoirs. The injected water breaks through into producing wells along high permeability channels or fractures and significantly decreases oil production. High water production has also generated several issues, including lift expense, reinjection of produced water, increased corrosion and scale, and increased environmental pollution, which eventually results in well shut-in [1,2]. So reducing water production of oil wells becomes an important emergency objective for mature oilfields [3-5]. 

Profile control and water shut off treatments are widely practiced to reduce water production and improve oil production [6,7]. Several techniques, including mechanical and chemical methods, are available for profile control and water shut off treatments. In general, mechanical methods, involving drilling multilateral wells and placing a bulkhead, are expensive and not affordable. Various chemicals methods, including injection of polymers, gel systems, foams, and particle systems, have been widely used for water shut off treatments in worldwide field trials [8-11]. However, a large amount of polymers are used in polymer flooding, which greatly increases recovery costs. In addition, polymer flooding is not suitable for serious heterogeneous reservoirs because of its weak profile control and water shut off capability. However, gel systems have inherent drawbacks, such as uncontrolled gelation time, uncertainness of gelling due to shear degradation, changes in gelant compositions, and dilution caused by contact with reservoir minerals and fluids. Moreover, foam injection technique has a short validity because of unsustained nitrogen or air sources. 

To overcome these problems, particle systems were synthesized in surface facilities. Among them, the most commonly used particle systems for water production control are preformed particle gels (PPG) [12-15], microgels [16-19], and dispersed particle gels (DPG) [20]. In general, PPG particles are prepared by emulsion polymerization with monomers and cross-linkers. Although PPG has overcome problems such as lack of control of gelation time and uncertainties due to the effect of adsorption and shear degradation, the size of PPG particles are usually in millimeters which cannot effectively be injected into conventional porous media. Also, the millimeter size of PPG particles limits their broad application in low permeability reservoirs. The microgels are usually prepared by shearing cross-linking with a coaxial cylinder viscometer or peristaltic pump. The shearing forces, generated from the coaxial cylinder viscometer or peristaltic pump, are conducted to the gelling solution, which makes it change into a discontinuous gel system and form microgels. However, due to the smaller displacement and lower production efficiency, it cannot meet the requirement of large-scale production, which limits the development of technology application in the oilfield. For easy preparation, easy injection and industry demands, DPG particles are proposed and prepared by the colloid mill with high speed shearing using cross-linked gel systems. However, cross-linked gel systems that have been used are only initiated between 60 °C and 85 °C, and the gelation time can vary from 3 days to a week [21]. As a result, the preparation period increases and causes additional energy consumption. So a gel system that can be cross-linked at low temperature is the most critical element in the preparation of DPG particles. Considering the simple preparation process and energy-saving, the gel systems used should have strong strength and be cross-linked within several hours at low temperatures. Gel systems based on multivalent cations, such as Al (III), Cr (III) or Zr (IV), can be cross-linked within several hours at low temperatures [9]. The gel strength of Al (III) gels is quite weak which is not suitable for DPG preparation. Moreover, gel systems based on Cr (III) cross-linkers cannot meet the current environmental regulations, because they are reported to be carcinogenic and environmentally unacceptable [22,23]. In the recent years, more interest has been concentrated on the zirconium gel system [24,25]. Zirconium is reported to have low toxicity and Zr (IV) can strongly interact with carboxylate groups to form complexes which are more stable than those formed by Al (III) or Cr (III) [26]. Thus, zirconium cross-linked polymer gels were used for the preparation of DPG particles in this study.

The aim of the present study was to investigate formation mechanisms of DPG particles with high speed shearing using cross-linked gel systems at room temperature. We primarily studied the gelation time, gel strength and microstructure of zirconium gel systems. The morphology, viscosity changes in the preparation process, size distribution and zeta potential distribution were investigated and a detailed preparation mechanism was discussed. The results of this study may serve as a reference for understanding the formation mechanisms of DPG particles. Through laboratory experiments, we expect the work can be further promoted and become applicable for water production control in mature oilfields.

## Materials and Methods

### Materials

Nonionic polyacrylamide (PAM) with the degree of hydrolysis 3.31 % and average molecular weight of 9,650,000 g/mol was provided by Yuguang Co. Ltd. China. Zirconium acetate as cross-linker was purchased from Zibo Co. Ltd. China. The salinity of brine was 400 mg/l, and was used in all experiments.

### Preparation of gels

A gelant solution was prepared by mixing the polymer solution and cross-linker at room temperature. The polymer solution was first diluted to the required concentration using brine. Then, the cross-linker was slowly dropped into the polymer solution and stirred to produce a uniform gelant solution. In this study, the cross-linking reaction was initiated at 30 °C. The gelation time and gel strength were determined by the pick hanging method and the breakthrough vacuum method, respectively [24]. 

### Preparation of DPG

The DPG was prepared by the high speed shearing method. 200 g of brine water and 200 g of bulk gel were simultaneously added to a colloid mill (JM-85 type, Shandong Longxing Instruments Ltd., China). Then, the colloid mill’s rotation speed was adjusted to 3000 rpm for 3-15 min at room temperature. Finally, the obtained solution was the DPG products.

### Characterization methods

#### Environmental scanning electron microscopy (ESEM) measurements

In this study, ESEM (Quanta 200 FEG, FEI Company Hillsboro, OR) was employed to observe the gelation microstructure. When the gel was formed in the ampoule, a drop of gel was directly placed on a covered ESEM grid. Pressure and temperature were initially set at 313 Pa to 455 Pa and 0 °C, respectively. Determinations were conducted at an accelerating voltage of 15 kV and a working distance range of 5 mm to 10 mm.

#### Scanning electron microscopy (SEM) measurements

SEM was used for the observation of DPG particles. A drop of the solution was placed onto a SEM grid (copper grid, 3.02 mm, 200 mesh). Then the sample was quickly transferred to a vacuum cup of liquid nitrogen and frozen at −80 °C for 2 hours. Subsequently, the sample was put into a lyophilizer for 24 hours with vacuum pressure ranging from 7 Pa to 8 Pa. After freeze-drying, DPG particles were investigated using a SEM (Hitachi S-4800) with an accelerating voltage of 3.0 KV.

#### Transmission electron microscopy (TEM) measurements

A JEOL 100CX-II TEM, operating at 100 kV, was used to investigate the samples. The samples for TEM analysis were prepared through freeze-drying as described above.

#### Atomic force microscopy (AFM) measurements

The microscopy of DPG particles was analyzed with a Nanoscope IVa MultiMode AFM (Digital Instruments, Santa Barbara, CA) in tapping mode and operated at a scanning speed of 1 Hz. Samples were prepared for AFM measurements by dropping a small amount of DPG solution onto a freshly cleaved mica surface for several minutes, and then gently dried with ultrapure nitrogen gas. Topographic and phase images were concurrently recorded under ambient conditions, at 512×512 pixel resolution, and integral and proportional gains 0.1-0.2 and 0.2-0.3, respectively.

#### Dynamic light scattering (DLS) measurements

The particle size distribution experiments were performed using a DLS measurement (Nano ZS90, Malvern Instruments Ltd., Worcestershire, UK). The maximum size range of this instrument is specified from 0.3 nm to 10 μm. The concentration of DPG samples were diluted with distilled water to 0.04% prior to the determination of the size distribution. Measurement adjustments were set as follows: 2 measurements with 3 runs each (10 sec), 25 °C, measurement angle 173° backscatter.

## Results

### Preparation of gels

#### Gelation performance

**Figure 1:**
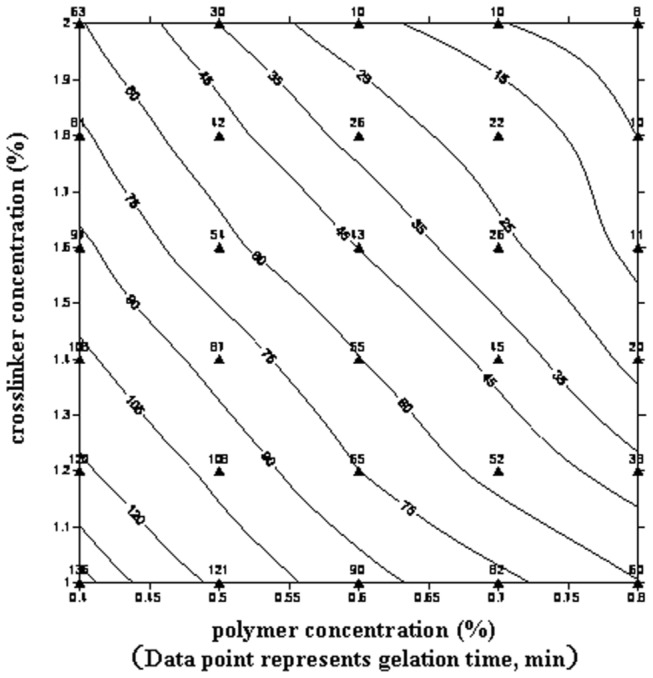
Contour map of gelation time.

**Figure 2:**
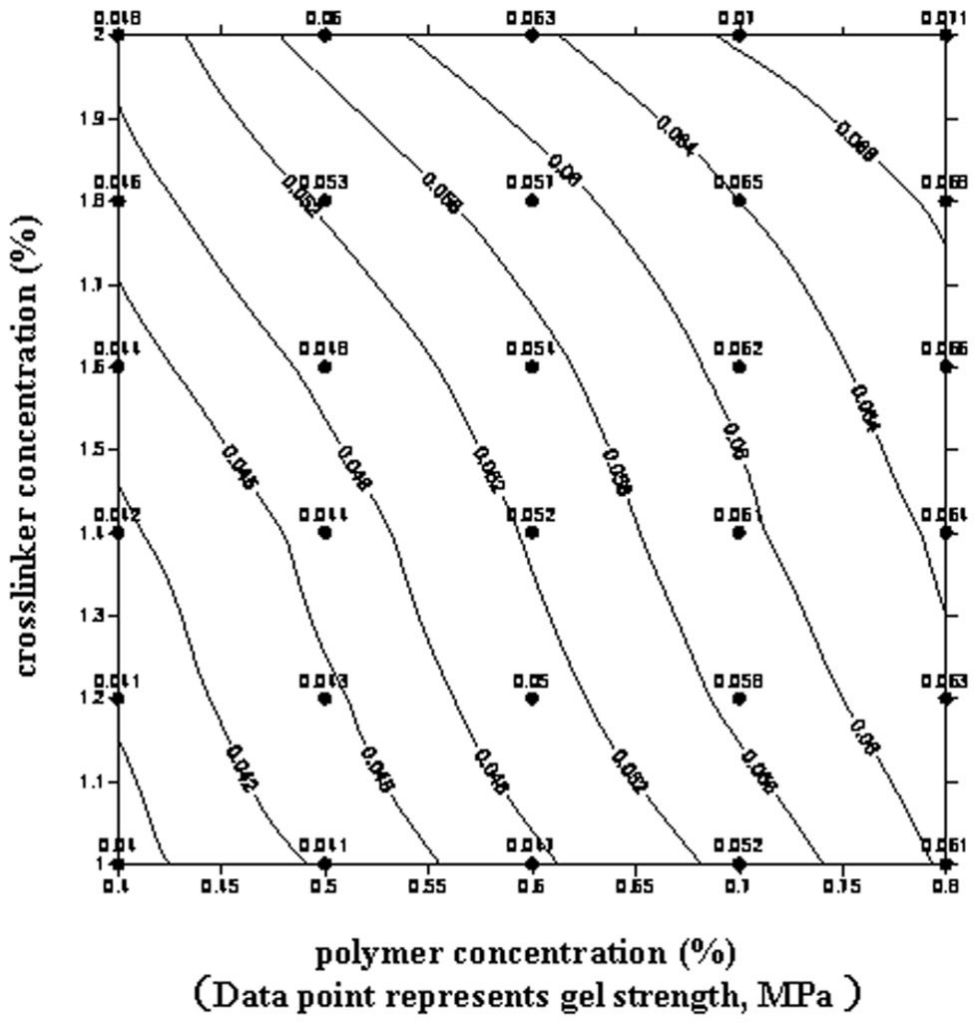
Contour map of gel strength.

The DPG particles were prepared using cross-linked gel systems through a simple high speed shearing method at room temperature. A gel system that can be cross-linked at low temperature is the most critical element in the preparation process. Considering the simple preparation process and energy-saving, the gel systems used should be cross-linked within several hours at low temperatures. In this study, the gel systems based on nonionic polyacrylamide and zirconium acetate cross-linker were investigated at 30 °C. The concentration of the polymer was varied from 0.4 % to 0.8 % while the cross-linker was varied from 1.0 % to 2.0 %. Figure 1 and Figure 2 show the contour map of gelation time and gel strength, respectively.

From Figure 1 and Figure 2, it can be seen that the polymer and the zirconium acetate can be rapidly cross-linked at 30 °C. The gelation time can be controlled from 8 min to 136 min by varying the polymer or cross-linker concentration whereas the gel strength can be adjusted to range from 0.04 MPa to 0.071 MPa. Higher concentrations of polymer or cross-linker produced from greater cross-linking densities in the gel networks increased the number of cross-linking sites (e.g., hydrophilic carboxylate groups, Zr(IV) complexes) [27,28]. Thus, the gel formation rate increased, leading to a decrease in the gelation time and an increase in gel strength. Based on gelation time and gel strength, an appropriate formula for preparation of DPG particles can be selected from the contour maps (Figure 1 and Figure 2). 

#### Microstructure of gels

**Figure 3:**
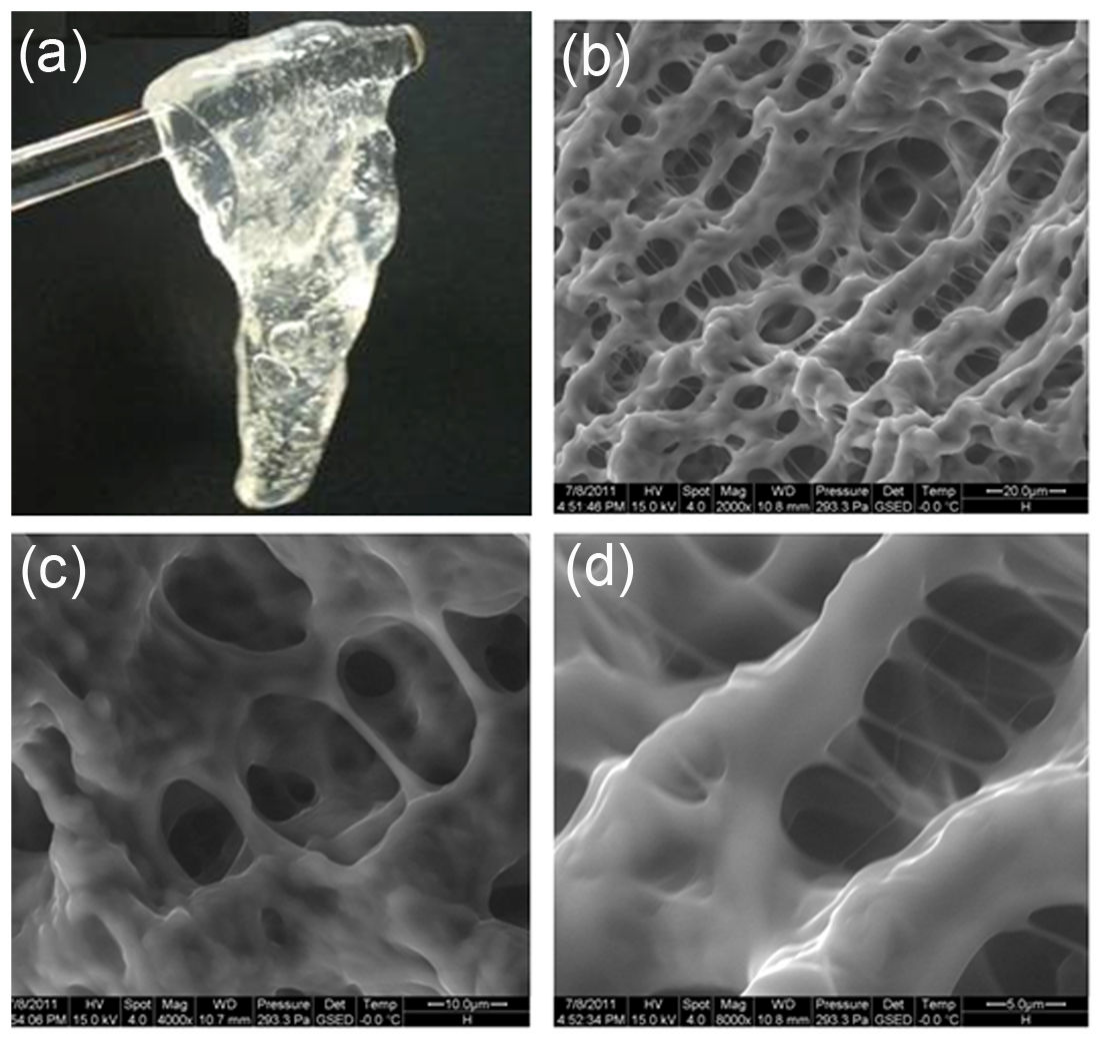
ESEM images of zirconium cross-linked polymer gels. (a) The bulk gel cross-linked by 0.6 % PAM and 1.6 % zirconium acetate; (b)-(d) Microstructure of the bulk gel at 2,000×, 4,000× and 8,000× magnification, respectively.

To better investigate the morphology of prepared DPG particles, ESEM was conducted to investigate the microstructure of bulk gel systems before shearing treatment [29]. Figure 3 shows the macrostructure and microstructure of zirconium gels.

Figure 3 (a) shows a colorless transparent gel obtained by reacting nonionic polyacrylamide with zirconium acetate cross-linkers. It is observed that the gels are easily deformable but not flowing (Figure 3 (a)). It can be considered a strong gel according to the pick hanging method [24].

Figure 3 (b)-(d) show typical ESEM images with different magnifications. Figure 3 (b) shows a 3-dimensional network structure of zirconium gel throughout the ESEM imaging procedure. The high magnification ESEM image showed that their surface possesses uniformly distributed structures with pore sizes ranging from several micrometers to approximately 10 μm (Figure 3 (c)). In the structure, the carboxylate group (–COO^-^) of the polymer is converted to a Zr (IV) complexation by a cross-linking reaction, incorporating hydrophilic groups into the chain bunch, which forms a 3-dimensional network. Furthermore, an obvious “skeletal structure” is formed (Figure 3 (d)), which plays a crucial role in enhancing the strength and stability of the gel system. In addition, this structure with numerous small pores may result in lower syneresis and great water holding capacity, thus promoting the stability of gel systems [30]. This structure is helpful to form stable DPG particles during the preparation process. 

### Preparation of DPG particles

To better understand the preparation technology of DPG particles, morphology, viscosity changes, particle size distribution and zeta potential were determined.

#### Morphology of DPG particles

The DPG particles were prepared by the high speed shearing method using zirconium cross-linked gel systems with the aid of a colloid mill at room temperature. The bulk gels (formula: 0.6% PAM + 1.6% cross-linker) were milled at 3000 rpm for 15 min. The obtained solution from the colloid mill was DPG products.

**Figure 4:**
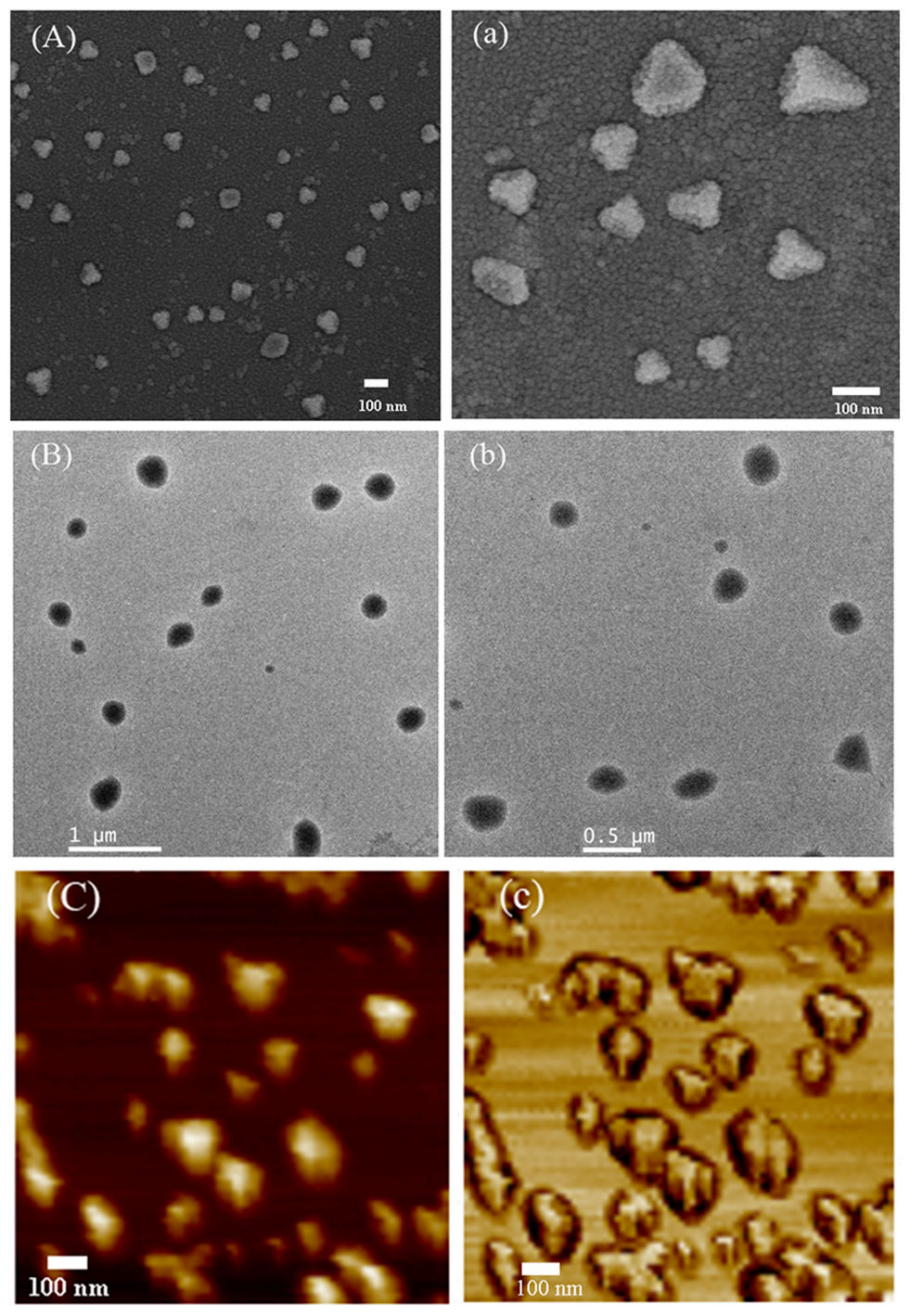
AFM, SEM and TEM images of DPG particles with Nano-size. (A): SEM images of DPG particles at 40,000× magnification; (a): SEM images of DPG particles at 100,000× magnification (B): TEM images of DPG particles at 4,000× magnification; (b): TEM images of DPG particles at 8,000× magnification; (C): AFM topographical images of DPG particles; (c): AFM phase images of DPG particles.

Figure 4 shows representative micrographs of DPG particles. In Figure 4 A) and a), morphology of DPG particles are clearly present. It can be seen that these particles are relatively uniform, characteristic of polygonal morphology. The largest size and average size of DPG particles were about 170 nm and 110 nm, respectively. In addition, the micrographs show DPG particles dispersed without aggregation. Figure 4 B) and b) show TEM images of DPG particles. It is evident that DPG particles still exist, but their morphology in TEM appears to be more regular, giving them a more oval appearance. This difference from SEM and TEM results probably arises from either small differences in the individual specimen preparations or different instrumental resolution. Further studies on particles morphology were also performed by using AFM measurements. From the images shown in Figure 4 C) and c), the largest particles were about 150-200 nm and several smaller DPG particles of 70 nm were also clearly visible. The results are almost consistent with the SEM and TEM analysis. These three analytical techniques confirmed the presence of DPG particles in the preparation process. The polygonal particle shape of DPG particles can be related to preparation techniques. In this research, the DPG particles were prepared using cross-linked gel systems through colloid mills at room temperature. Colloid mills are rotor-stator systems that can be used to reduce particle size distribution of liquid dispersions or solid dispersions. When bulk gel is pumped through a narrow gap that is formed by the rotating inner cone and the stationary outer cone, the shearing forces, generated from the relative movement between the stator and rotor, are conducted to the bulk gel, which makes the bulk gel change into small particles. In addition, the surfaces of rotor-stator systems are not smooth, but roughened and toothed, which increases wall friction and reduces slip, and then forms polygonal particles.

#### Viscosity changes in preparation process

**Figure 5:**
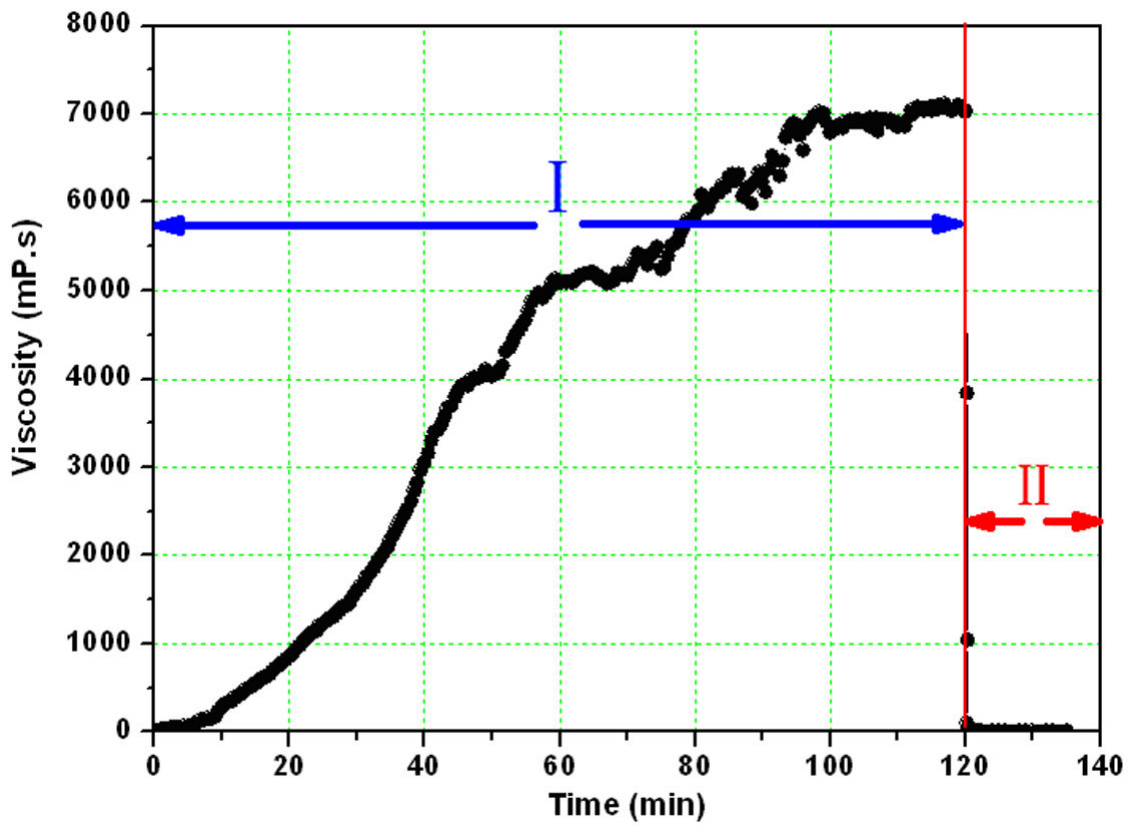
Viscosity changes in the whole preparation process. I: Viscosity changes during the bulk gel cross-linking reaction (formula: 0.4 % PAM + 1.6% cross-linker, the cross-linking time lasts from 0 min to 120 min) ; II: Viscosity changes during the DPG particle preparation process (the shearing time lasts from 120 min to 135 min).

The changes of viscosity are a characteristic feature in the DPG particles preparation process. Thus, it is important to investigate viscosity changes that can help to gain a better understanding of the preparation mechanism. Rheological measurements were carried out to investigate the viscosity changes at low shear rate (1s^-1^, under this shear rate, the gel can be formed without adverse effects). Figure 5 shows viscosity changes in the entire preparation process at 30 °C.

As shown in Figure 5, the viscosity slowly rises at the beginning. Then, the viscosity increases more rapidly and finally becomes stable, indicating the gelation. The whole process lasts for about 120 minutes and the viscosity eventually reaches 7040 mPa·s. However, when adding the bulk gel to a colloid mill rotating at 3000 rpm and milling for 15 min, the viscosity sharply decreases and eventually reaches 5.2 mPa·s. The obtained solution from the colloid mill was the final DPG products. As can be seen from the changes in viscosity, the whole preparation process can be divided into two successive steps: the bulk gel cross-linking reaction period and the DPG particle preparation period. 

Step I: The bulk gel cross-linking reaction period. The viscosity changes in this step have been elaborated in our previous research [24]. According to viscosity changes in the cross-linking reaction process, it can be divided into three successive steps: the induction period, the rapid cross-linking period, and the stabilization period. Then a 3-dimensional gel network structure is formed, resulting in no further viscosity increase [31].

**Figure 6:**
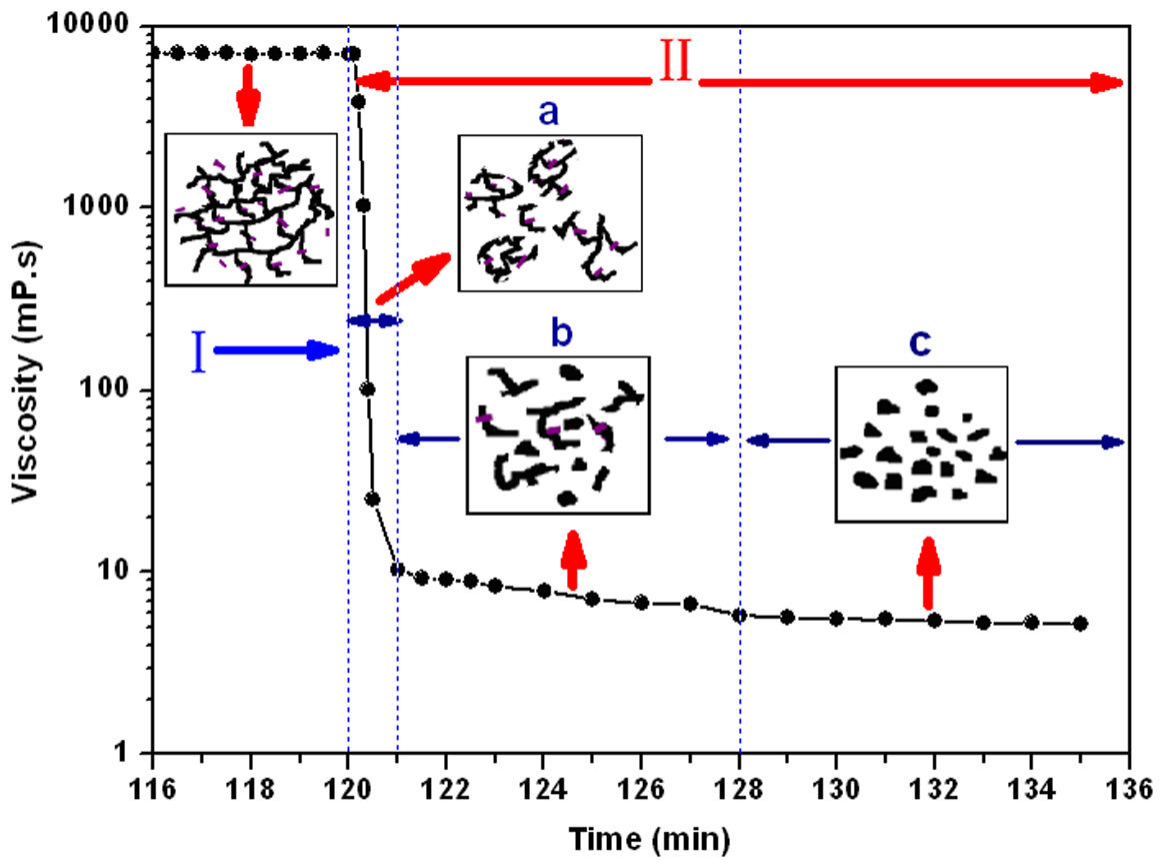
Viscosity changes during the DPG particle preparation process. a) Broken rounded period; b) Further milling period; c) Particle homogeneous period.

Step II: The DPG particle preparation period. Figure 6 further shows viscosity changes in this period. A significant decrease in the gel viscosity appears. This preparation process can be divided into three successive steps: the broken rounded period (Figure 6 a), the further milling period (Figure 6 b), and the particle homogeneous period (Figure 6 c). 

Period (a): Broken rounded period. It is interesting to note that the viscosity sharply decreases in the broken rounded period. The viscosity decreased from 7040 maP·s to 10 maP·s when adding the bulk gel into a colloid mill rotating at 3000 rpm and milling for only 1 min. At the beginning of milling, the bulk gel systems retained their gelled state and mechanical consistency, so the systems maintain the highest viscosity values. However, when the bulk gels pass through the colloid mill, high shearing stress is conducted to the bulk gel system and gels are broken into coarse particles, then causes a sharp reduction in viscosity (Figure 6 a).

Period (b): Further milling period. As the milling continues, the coarse particles are further reduced and the size distribution of the produced particles widely varies from relatively coarse to fine because of the wall friction. As a result, the weak interaction between the particles can be easily destroyed by high shearing force, which induces a further viscosity reduction. 

Period (c): Particle homogeneous period. The viscosity can no longer decrease when the shearing time is increased. That is because the particles become uniform and smaller because of long-term shearing, so they can be easily pass through the narrow gap that is formed by the rotating inner cone and the stationary outer cone. As a consequence, the shearing force almost has no effect on the particles size reduction until it forms a stable system, resulting in no further viscosity decrease.

#### Size distribution

**Figure 7:**
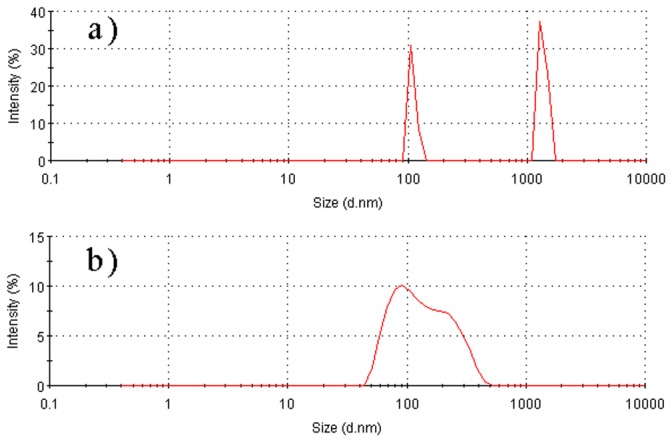
Particle size distribution of DPG particles determined by dynamic light scattering. a) the bulk gels (formula: 0.6 % PAM + 1.6% cross-linker) were milled at 3000 rpm for 6 min; b) the bulk gels were milled at 3000 rpm for 15 min.

The size distribution of DPG particles was determined by DLS measurements. Figure 7 shows the mass particle size distribution of DPG particles which were milled at 3000 rpm for 6 min and 15 min, respectively. As shown in Figure 7 a), the intensity distribution of the hydrodynamic size of DPG particles has two peaks. The first peak (about 60.9 % of the particle intensity) has an average size of about 1359 nm with a peak width of 98.57 nm, whereas the second peak (about 39.1 % of the particle intensity) has an average size of about 109.3 nm with a peak width of 6.837 nm. Thus, the average DPG particle size is around 804 nm with a polydispersity index (PDI) of 0.7 and a standard deviation of 18.9563. The results suggest that DPG particles are relatively non-uniform when only milling for 6 min. However, there is only one peak in the particle size distribution curve after another 9 min of milling under the conditions stated above (Figure 7 b). It suggests that the prepared particles tend to be uniform. Most particles range from 80 to 200 nm, and the average size distribution of particles decreases from 804 nm to 109 nm as the shearing time increases from 6 min to 15 min. In addition, the polydispersity index (PDI) and standard deviation are also decreased to 0.247 and 1.885, respectively. This gives evidence that the size distribution of particles has become significantly uniform with increasing shearing time. However, when sheared for a short time (6 min), the bulk gel is also divided into particles under the shearing force. Both the coarse particles and fine particles are simultaneously formed at this moment, but the coarse particles account for the majority in the solution, so the particle size distribution curve has two peaks. As the shearing proceeds, the shearing forces continue to be conducted on the coarse particles, thus making the particles fine and uniform, resulting in a narrow particle size distribution (Figure 7 b). In addition, the observed hydrodynamic diameters (Figure 7 b) are almost consistent with those observed in the corresponding SEM, TEM and AFM images.

#### Zeta potential

**Figure 8:**
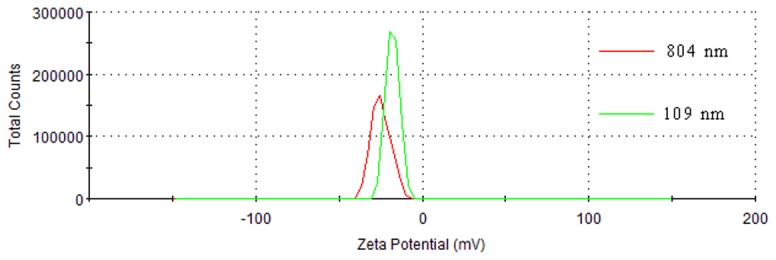
Zeta potential distribution of DPG particles with different sizes.

The zeta potential serves as an important parameter in characterizing the electrostatic interaction and properties between particles in dispersed systems [32-34]. The zeta potential has an important impact on the stability of particles solution. In the present research, the zeta potential was determined using a zetasizer instrument (Nano ZS90, Malvern Instruments Ltd., Worcestershire, UK) at 30 °C under a pH of 5.6. Figure 8 shows the zeta potential of DPG particles with two different particle sizes (804 nm, 109 nm).

From Figure 8, the absolute zeta potential tended to increase whereas particle size appeared to exhibit a decreasing trend. But the DPG particles with two different particle sizes have a similar surface charge. Under weak acid conditions, the two DPG particles of 109 nm and 804 nm are negatively charged with an average of -24.8 mV and -17.8 mV, respectively. It appears to suggest that DPG particles are not of exceptional quality with respect to their existence in the form prepared. In general, a stable colloidal system is recognized as having an absolute value of zeta potential over 30 mV, as the surface charge prevents aggregation of particles [35]. But for our DPG particle systems, the DPG particles have a relatively small negative charge, which is more useful for injection into the formation. When DPG particles were dispersed in water, the electrostatic repulsion between them caused the particles to repel each other and prevented aggregation, resulting in slightly stable colloidal systems. However, the metals ions (Na^+^, Ca^2+^, et.al) from the formation water will neutralize the negative charges of the DPG particle surface when injecting the particles into the formation. Thus, a decrease in the charge will result in a reduction in the electrostatic repulsions. As a consequence, a large amount of DPG particles bind into tiny microaggregates. Microaggregates, in turn, combine to form larger aggregates in the formation. Then the larger aggregates bridge across the pore throats and reduce permeability of reservoir cores.

### Formation mechanism

**Figure 9:**
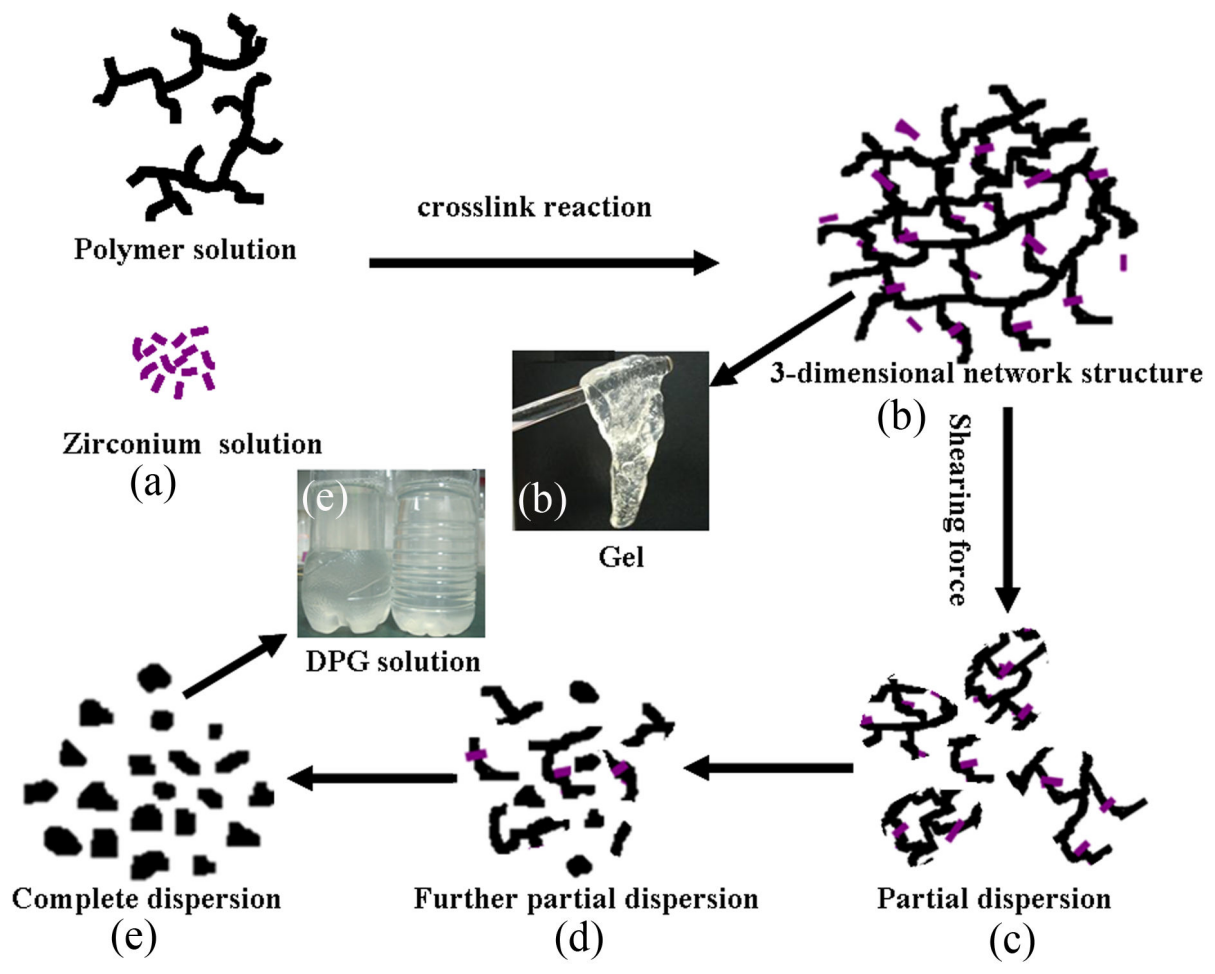
Proposed formation mechanism of the dispersed particle gel.

Based on the above research, a possible preparation and formation mechanism of DPG particles has been proposed and is shown in Figure 9. The whole preparation process can be divided into two major stages: the bulk gel cross-linking reaction period and the DPG particle preparation period. At the beginning of bulk gel cross-linking reaction period, the carboxylate group (–COO^-^) of the polymer is converted to a Zr (IV) complexation by a cross-linking reaction, forming a bulk gel with a 3-dimensional network (Figure 9 (b)). The entire process from the cross-linking reaction to the bulk gel formation can be completed within 2.5 hours at 30 °C, which can help ensure the efficiency of the preparation process. When adding the bulk gel into a colloid mill, the subsequent "DPG particle preparation period" was conducted. The high shearing forces are conducted to the bulk gel, which makes it change into a discontinuous gel system (Figure 9 (c)). This process can be rapidly completed within about 1 min, and the bulk gel is only partial dispersed. Subsequently, the discontinuous gel system continues to pump through a narrow gap that is formed by the rotating inner cone and the stationary outer cone. The shearing force further crushes the discontinuous gel system forming a large number of coarse particles in the systems (Figure 9 (d)). This stage usually lasts for 5-7 min. As the milling continues, the size distribution of coarse particles widely varies from relatively coarse to fine. The large centrifugal force caused by the relative movement between the stator and rotor, can make coarse particles slide on the stator surface. In turn, this sliding on the stator surface will produce a frictional force, which produces fine and uniform particles. In addition, the surfaces of rotor-stator systems are not smooth but roughened and toothed, which further increase wall friction and reduces slip. After another 3-7 min milling, the polygonal DPG particles are formed and then uniformly dispersed in the solution (Figure 9 (e)). The above results show that the whole preparation process can be completed within 3 h at room temperature. It can be an efficient and energy-saving technology for preparation of DPG particles.

## Discussion

In the present report, the DPG particles were successfully prepared using a polymer gel within 3 h at room temperature. The results show that the polymer gel was a key factor for the DPG particle preparation technology. Although previous studies showed that phenolic resin cross-linked polymer gels were successfully used in oilfields, the high cross-linking temperature (60 °C-85 °C) and long gelation time limit the application in DPG particle preparation technology [21]. Due to the simple preparation process and energy-saving, low gelation time, strong gel strength and low cross-linking temperature are the necessary conditions to choose polymer gels. We have shown that the zirconium acetate cross-linked polymer gels with strong gel strength can be cross-linked within 2.5 h at 30 °C. ESEM studies further confirm the gel systems have a compact structure which is helpful to form stable DPG particles.

Viscosity changes are the most important characteristic in the preparation process. An increase in viscosity indicates that the bulk gel was formed, whereas the viscosity decreases when the shearing force was conducted on the bulk gel systems. From the analysis of the viscosity changes, the bulk gel cross-linking reaction period and DPG particle preparation period were the two major stages in the whole preparation process. Chemical reaction makes the gelling solution form bulk gel systems while the physical shearing is the driving force for the formation of DPG particles. It is the DPG particle’s formation mechanism. This technique overcomes some of the limitations of conventional methods [12,16,19], such as complicated operation, low production efficiency and high cost. Using this technology, nano-sized DPG particles with negative charge were prepared. The size distribution and negative charge were conducive to inject and aggregate in the formation. Additionally, the increasing shearing force reduces the particle size distribution, and then decreases zeta potential. So we can control the shearing force to prepare different DPG particles. 
